# The role of traditional healers and barriers to collaboration with biomedical providers in mental health service delivery in Wakiso district, Uganda: a qualitative study

**DOI:** 10.1186/s12913-026-14170-5

**Published:** 2026-02-19

**Authors:** David Musoke, Sarah Nalinya, Grace Biyinzika Lubega, Michael Obeng Brown, Linda Gibson

**Affiliations:** 1 Department of Disease Control and Environmental Health, Makerere University School of Public Health, P. O. Box 7072, Kampala, Uganda; 2 Institute of Health and Allied Professions, School of Social Sciences, Nottingham Trent University, Nottingham, NG1 4FQ UK

**Keywords:** Mental health, Traditional healers, Biomedical practitioners, Collaboration, Health services, Uganda

## Abstract

**Background:**

Mental health remains a low priority issue in many low- and middle-income countries where both biomedical practitioners and traditional healers provide essential services. While the collaboration between these two types of providers is essential for comprehensive care, their integration within Uganda’s mental health service delivery system remains insufficient. This study therefore explored collaboration between biomedical and traditional providers in mental health service delivery in Wakiso district, Uganda.

**Methods:**

We conducted a qualitative study that employed key informant interviews to collect data. Thirty-four (34) key informants including 15 biomedical practitioners, 11 traditional healers, and 8 national level stakeholders such as policy makers participated in the study. All interviews were audio recorded and transcribed. Thematic analysis was conducted with the support of NVivo 2020 (QSR International).

**Results:**

Two main themes emerged from the study: the role of traditional healers in the mental health service delivery; and challenges affecting collaboration of traditional healers and biomedical practitioners. The study established that although not fully recognised, traditional healers played an important role in mental health through easing access to health services, affordability of their services, and the trust that communities had in traditional medicine. Non-inclusive mental health policy, power imbalances, and limited funding for mental health research were the main challenges identified regarding the collaboration between traditional healers and biomedical practitioners in mental health service provision.

**Conclusions:**

Traditional healers are actively involved in mental health service provision in Uganda. Collaboration between traditional healers and biomedical practitioners could be enhanced by various stakeholders including policy makers recognising the contribution of traditional medicine, ensuring inclusive mental health policies, and increased resources for mental health research.

**Supplementary Information:**

The online version contains supplementary material available at 10.1186/s12913-026-14170-5.

**Table:** 

Text box 1. Contributions to the literature
• Collaboration between biomedical and traditional providers is essential for comprehensive mental health care particularly in low-income settings.
• There is limited evidence on the collaboration between biomedical practitioners and traditional healers in mental health service delivery in Uganda and other parts of the world.
• Policy makers and other stakeholders should enhance collaboration between biomedical practitioners and traditional healers by recognising the contribution of traditional medicine, ensuring inclusive mental health policies, and increased resources for mental health research.

## Introduction

Mental health is a key component of the Sustainable Development Goals (SDGs) as part of the global agenda to increase access to equitable health care [1, 2]. However, mental health care is still largely a low priority issue at all levels of health service delivery in many low- and middle-income countries (LMICs) including Uganda. This creates a gap where a large proportion of people with mental health conditions are not aware of their situation [3]. In addition, many LMICs inclusive of Ugandan communities, understand mental health conditions through spiritual, social or ancestral frameworks that differ from biomedical models [4–6]. Good mental health in itself is not only valuable for living, but also a key determinant for physical health [7]. Poor mental health is associated with risky health behaviour such as substance abuse, violence, physical inactivity, and poor diet, which can lead to chronic diseases [8–10]. At community level, poor mental health can result in stigma and discrimination, increased health expenditure, loss of school and workdays, and social drift into poverty [6, 11–13]. Uganda has a high prevalence of mental health conditions estimated at 22.9% and 24.2% among persons below and above 18 years respectively [14]. The most common conditions include post-traumatic stress disorder (PTSD), depression, anxiety and schizophrenia. A detailed description of Uganda’s mental health burden can be found elsewhere [15–17].

Mental health service providers in Uganda are varied including biomedical practitioners (such as psychiatrists, psychologists, doctors, clinical offices, nurses, social workers, and pharmacists) [18] and traditional healers (including herbalists and spiritualists) [19, 20]. Both biomedical practitioners and traditional healers offer mental health services such as counselling, support and treatment of mental health conditions. While biomedical providers offer mental health services which are supported by widely known evidence, traditional healers provide health services in line with local belief systems and knowledge [20–22].

The importance of access to mental health services for all is emphasized by the SDGs, which uphold mental health as a priority for which service coverage indicators are important [1]. However, LMICs such as Uganda are still grappling with challenges such as major inequalities in resource distribution for mental health notably staffing and in-patient beds [5, 23]. For example, the availability of mental health care services is much higher in urban areas, yet this is not where the minority of the population live [5, 23, 24]. As of 2022, Uganda had only 53 psychiatrists—approximately one psychiatrist per 1 million population [23]. The gap in mental health service provision in the public sector is normally bridged by traditional health providers. Since biomedical practitioners and traditional healers are both involved in mental health service delivery, close collaboration between the two groups could lead to improved outcomes among the population [4]. However, barriers to a good working relationship between the two categories of providers exist in Uganda because of negative stereotypes, and different philosophies, as well as varying approaches to treatment [16, 18, 20, 25]. For example, previous studies revealed that traditional healers were more likely to refer mental health patients to other traditional healers instead of clinicians [25, 26]. This is because many traditional healers believe that biomedical practitioners undermine their practice and would not want to collaborate with them [18]. Additionally, many traditional healers are often sidelined by biomedical practitioners during their practice [17].

The World Health Organization (WHO) endorsed the involvement of traditional healers in mental health and considers them as a viable solution in achieving universal health coverage [27]. Indeed, the WHO Comprehensive Mental Health Action Plan 2013–2030 highlights the importance of collaboration between biomedical providers and traditional healers to ensure better mental health outcomes in communities [28]. However, this collaboration has not yet been streamlined in the provision of mental health services in Uganda [20]. For example, the current legislation and policies in Uganda including the Mental Health Act (2018) do not explicitly recognise traditional healers as providers of mental health care. Therefore, there is no clear indication of how mental health providers in biomedical and traditional settings should collaborate [29]. Although WHO recommends integration of traditional medicine into national health systems [30], evidence from Uganda shows persistent resistance and minimal collaboration between biomedical and traditional healers [4, 20]. More research is required to justify the need for integration, particularly in mental health [4]. Indeed, there is need for more evidence on impartial insiders’ perspectives on working together between biomedical professionals and traditional healers in mental health. This study therefore explored collaboration between biomedical and traditional providers in mental health service delivery in Wakiso district, Uganda.

## Methods

### Study design and setting

The study used qualitative methods, specifically key informant interviews (KIIs). A qualitative approach was deemed appropriate for the study to be able to generate in-depth contextual insights and experiences of collaboration between biomedical and traditional providers. Wakiso district, located in Central Uganda, was purposively selected as a suitable study site due to having a good mix of urban, peri-urban and rural communities. Mental health conditions were known to exist in the district which are exacerbated by environmental and societal stressors [13]. In addition, Wakiso district is the most populated district in Uganda, while its capacity to manage mental health illnesses is limited. Indeed, the district faces challenges in mental health service delivery regarding human resources, finances and supplies [31]. Specifically, Busiro South Health Sub District, which consists of Kajjansi, Kasanje and Katabi town councils, and Bussi sub-county, was involved in the study. Busiro South has vast rural and urban communities and a population of 244,309 [32]. The main economic activities carried out in the area include agriculture, small scale trade, brick making and sand mining. The health sub district had 8 government health facilities and several private facilities including clinics. Community health workers (CHWs), locally referred to as village health teams, also exist in the area and are the first contact of most of the community with the health system. In the Ugandan context, other health cadres are not included in the definition of CHWs.

### Study participants and data collection

In order to gain an understanding of the collaboration between biomedical and traditional mental health service providers, the study participants included several stakeholders at national, district and community levels. These participants were purposively selected due to their knowledge and practice either in traditional medicine, biomedical mental health, or health policy in Uganda. The key informants were broadly divided into three categories of: biomedical providers; traditional healers; and national and sub-national stakeholders. The 15 biomedical health service providers included psychiatrists, psychologists, psychiatric nurses, doctors, clinical officers, general nurses and CHWs. The 11 traditional health service providers included spiritualists, diviners, religious leaders, and herbalists. These individuals often work within communities to provide alternative mental health care for the population. In addition, leaders of the associations of different traditional health service providers were involved. The 8 national and sub-national participants included policy makers, implementing partners and other national level stakeholders including representatives from WHO, Ministry of Health (MOH), Wakiso District Health Office, non-governmental organisations (NGOs) supporting mental health, and mental health researchers. Any individuals who were not actively involved in biomedical or traditional mental health practice, as well as mental health policy, implementation or research at the time were excluded from the study.

The KIIs generated insights from key stakeholders concerning mental health service provision and the collaboration between biomedical practitioners and traditional healers. KII guides for the 3 categories of participants were developed with reference to existing literature on mental health both locally and globally (see study tools’ supplementary file). In addition, a KII guide specifically for CHWs was developed given their unique role in the community. The KII guides for traditional healers and CHWs were translated from English to *Luganda*, the local language most commonly used in Wakiso district. Data collection was conducted between 27th September 2021 and 21st January 2022 by two research assistants with vast experience in qualitative research. The research assistants, who had bachelors degrees in a health related field, were trained and oriented on the study by the principal investigator to ensure they were well versed with the tools before data collection. The KIIs were conducted at the preferred place of the participants which was private (most suitably at their workplaces / stations particularly in offices / health facilities / community setting). However, some interviews were conducted virtually via Zoom due to local COVID-19 travel restrictions at the time. All the tools were pretested in Wakiso district among a group having similar characteristics with the participants but were not involved in the main study. Following pretesting, revisions were made to the tools that made them well suited for data collection.

### Sampling

A mix of purposive and snowball sampling strategies were employed to obtain participants from the different categories. The selection of key informants, particularly biomedical providers, policy makers, and national level stakeholders, was purposively based on those who were involved and experienced in mental health service delivery. Snowball sampling was specifically useful for obtaining other traditional healers in the community. This strategy was employed as it was challenging to get an adequate number of traditional healers through other approaches. The study involved 34 participants from the different categories at which data saturation was reached. Data saturation was realised when no new information was emerging from the interviews being conducted. The key informants were obtained at the national level (such as MOH, NGOs and researchers) as well as the sub-national level (such as Wakiso district), and community level (including traditional healers and CHWs).

### Data management and analysis

Data from the KIIs was audio recorded in either English or *Luganda*, depending on the participants. For example, data from the MOH officials, biomedical service providers and researchers was collected and recorded in English, while for the traditional healers and CHWs was collected in *Luganda*. All recorded data was transcribed verbatim by one of the research assistants and proof-read by another to ensure accurate record of the interviews. Once the transcripts were verified, those in *Luganda* were translated to English by the research assistants for analysis. Data analysis was conducted thematically [33] by two members of the research team with experience in qualitative data analysis (SN – MPH and GBL – MPH) in NVivo 2020 (QSR International). The two team members read through the transcripts and developed codes which they discussed and revised with the principal investigator (DM) who helped to resolve any disagreements. The codes were defined, and several quotes represented by the different codes were highlighted to develop a code book. Intercoder reliability was achieved which involved comparing, discussing, and refining the generated codes. Codes that were linked or those that covered a similar subject were grouped to form sub-themes. Related sub-themes were then combined into overarching themes which were reviewed and refined by the research team. The final themes are the major findings emanating from the study.

## Results

Findings from the study that involved 34 key informants (Table 1) are presented under two themes: role of traditional healers in providing mental health services; and challenges to collaboration between biomedical practitioners and traditional healers. The role of traditional healers is presented under three sub-themes of accessibility, affordability, and trusted alternative to conventional mental health care. Three sub-themes on the challenges to collaboration were non-inclusive mental health policy framework, traditional medicine considered inferior, as well as limited funding for research (Table 2).

**Table 1:** Study participants

Participant number	Participant role	Category
1	CHW 1	Biomedical providers
2	CHW 2	Biomedical providers
3	CHW 3	Biomedical providers
4	CHW 4	Biomedical providers
5	CHW 5	Biomedical providers
6	CHW 6	Biomedical providers
7	Religious leader (Anglican)	Traditional healers
8	Herbalist 1	Traditional healers
9	Traditional healer 1	Traditional healers
10	Biomedical healthcare provider (lower-level government facility) 1	Biomedical providers
11	Biomedical healthcare provider (lower-level government facility) 2	Biomedical providers
12	Traditional healer 2	Traditional healers
13	Religious leader (Moslem)	Traditional healers
14	Herbalist 2	Traditional healers
15	Religious leader (Pentecostal)	Traditional healers
16	Herbalist 3	Traditional healers
17	Biomedical healthcare provider (lower-level government facility) 3	Biomedical providers
18	Biomedical healthcare provider (lower-level government facility) 4	Biomedical providers
19	Traditional healer 3	Traditional healers
20	Traditional healer 4	Traditional healers
21	Traditional healer 5	Traditional healers
22	Psychiatric nurse (lower-level government facility) 1	Biomedical providers
23	Psychiatric nurse (lower-level government facility) 2	Biomedical providers
24	Mental health researcher 1	National and sub-national stakeholders
25	Psychosocial support personnel	National and sub-national stakeholders
26	Mental health researcher 2	National and sub-national stakeholders
27	Psychiatric nurse (lower-level government facility) 2	Biomedical providers
28	Psychiatric nurse (lower-level government facility) 3	Biomedical providers
29	Mental health practitioner (NGO) 1	National and sub-national stakeholders
30	Mental health practitioner (NGO) 2	National and sub-national stakeholders
31	Mental health specialist (national government referral hospital)	Biomedical providers
32	Mental health policy maker 1	National and sub-national stakeholders
33	Mental health policy maker 2	National and sub-national stakeholders
34	Mental health researcher 3	National and sub-national stakeholders

**Table 2:** Themes and sub-themes on role of traditional healers and challenges to their collaboration with biomedical practitioners

Theme	Sub themes
Role of traditional healers in providing mental health services	Accessibility
	Affordability
	Trusted alternative to conventional mental health care
Challenges to collaboration between biomedical practitioners and traditional healers.	Non-inclusive mental health policy framework
	Traditional medicine considered inferior
	Limited funding for research

### Role of traditional healers in providing mental health services

#### Accessibility

The participants, particularly biomedical providers, revealed that traditional healers were important providers of mental health services because they were strategically located within communities and understood the local cultures and contexts of their conditions, hence making them more readily accessible. It also emerged that most traditional healers operated from within walking distance of many households hence making it easy for clients to approach and access them.

*“The majority of first mental health care is from alternative care providers. This is because they are more locally accessible and acceptable*,* as well as fit within the communities’ cultural understanding and interpretation of what mental illness is.”* Key informant 31, biomedical service provider from the national mental health referral hospital

In addition, the participants claimed that traditional healers being more accessible to the population was also due to their being more in number in comparison with biomedical providers. They added that bureaucracy involved in accessing biomedical practitioners in Uganda also made them less accessible to the population, making people to resort to traditional healers who required fewer processes while providing mental health care. Recommendation of traditional healers by family and friends also increased their visibility hence accessibility within communities.

*“Our health system is structured such that mental health services are still highly centralized*,* with very few mental health specialists. So*,* for example*,* in certain areas*,* for one to get good mental health care*,* they will have to go to the regional referral hospital*,* which is out of reach for a lot of people and their caregivers. In addition*,* the bureaucracy involved in getting mental health care is too much because of the few mental health service providers. On the other hand*,* traditional healers are very many*,* they are everywhere*,* more visible and accessible.”* Key informant 24, mental health researcher

## Affordability

Participants revealed that the community sought mental health services from traditional healers because they perceived them to be more affordable than biomedical practitioners. This finding on affordability was both in terms of overall cost of services, as well as the fact that charges from traditional healers were negotiable and could be paid in instalments.

*“We traditional healers understand our communities’ financial situation and therefore consider them on a humanly basis. We therefore charge less for our services*,* and the money does not have to be paid at once. I have seen people stuck in hospital due to unaffordable medical bills*,* and this discourages them from seeking mental health care from such facilities.”* Key informant 19, traditional healer

### Trusted alternative to conventional mental health care

From the study findings, the community had trust in traditional healers to provide effective care for mental health conditions, hence usually used them as the first line of treatment. This finding, which emerged from both biomedical providers and traditional healers, was partly because of the positive testimonies from other community members following using the services of traditional healers.

*“According to the history we take from patients*,* they usually first go traditional healers before coming to us. I think almost 90% first go to traditional healers*,* mainly due to recommendation from their friends and family hence the increased trust in their services.”* Key informant 18, biomedical service provider from a lower-level health facility

Some key informants reported that it was common for patients to concurrently use traditional medicine while also seeking biomedical care for mental health conditions. This finding was partly due to the perception that biomedical treatment cannot cure all diseases including mental illnesses which are at times thought to be related to spirits. The participants also said that many community members perceived traditional medicine to have less undesirable side-effects compared to biomedical care hence the trust and confidence in traditional health services.

*“What I am sure about is that modern medicine cannot treat the power of spirits. If it can treat such cases*,* I haven’t heard anyone say that modern medicine has helped them recover from ancestral spirits and then they write down the mental health condition as it is arising from such a source. However*,* a person can be on treatment with modern medicine as well as traditional remedies simultaneously to try and get better.”* Key informant 13, religious leader

### Challenges to collaboration between biomedical practitioners and traditional healers

#### Non-inclusive mental health policy framework

It emerged from the study that non-inclusive policies were one of the major hindrances to collaboration between biomedical practitioners and traditional healers. For example, it emerged that the various mental health policy documents for Uganda hardly recognised traditional healers as providers of mental health services. In addition, many traditional healers thought that they were not fully regulated by the government which made it difficult to standardise their services. The participants added that it would be difficult for traditional healers to collaborate with biomedical practitioners due to the absence of guidelines or a framework for the collaboration.

*“I think we need to come up and be genuine and express the will to recognize them [traditional healers] and then probably have that kind of arrangement to bring them on board. But as per today*,* the biggest challenge is with the regulatory mechanism and also defining their roles. For example*,* who will regulate traditional healers’ practice and to what extent? Although there is a brief Traditional and Complementary Medicine Policy*,* the role of traditional healers is not well stipulated and consolidated by the Mental Health Act.”* Key informant 24, mental health researcher

### Traditional medicine considered inferior

Some key informants revealed that biomedical practitioners consider mental health services provided by traditional healers to be inferior, ineffective and sometimes harmful to patients. This was mainly due to limited documented research in Uganda’s local herbs and spirituality. In addition, the participants also cited unstandardized and unregulated operation of traditional healers as a factor for their perceived inferiority to biomedical practitioners.

*“Those people [traditional healers] usually make patients with mental illness worse. For example*,* someone with mild depression may go to a shrine*,* and they are kept there until their condition worsens. That is why medical practitioners have a negative attitude towards them. Because spirituality is not a well-documented science*,* many traditional healers also exploit the patients and conduct illegal activities such as physical torture*,* sexual abuse*,* and holding the patients against their will.”* Key informant 34, mental health researcher

Traditional healers reported that biomedical practitioners could not refer mental health patients to them due this perceived inferiority of their services. This finding was therefore seen to make collaboration between the two groups challenging if they had perceived power differences.

*“Unfortunately*,* we don’t have any formal collaboration. What we have is ideally very informal and rudimentary because in some instances*,* the traditional healers are the first point of contact for patients. When traditional healers realize that they cannot handle patients*,* they refer them to the mental health units at the health facilities and indeed they do some referral. But we do not do any referrals to them*,* because it is not considered ethical in the medical profession. It is mainly a one-way referral.”* Key informant 24, mental health researcher

#### Funding for research

Participants reported that there was generally limited government funding allocated to mental health research in Uganda. As a result, there was limited evidence on the possibility of collaboration between biomedical practitioners and traditional healers, making it an unexplored area in Uganda.

*“One of the biggest challenges is that mental health in Uganda and generally around the world is underfunded. Mental health is being side-lined in terms of funding as people who would rather fund something else such as surveillance and other research fields but not mental health yet there is not much to do with no funding. That is why research on how to improve mental health in Uganda is currently scanty.”* Key informant 26, mental health researcher

Traditional healers reported that there were limited public funds allocated to research in traditional medicine, and most research in the field mainly relied on private funding. This made it challenging to have standardised training and operating procedures for traditional healers.

*“It is very rare that a traditional healer is funded by government to conduct research. Most of us use external funding or our personal funds to do our research and this makes it very difficult*,* and discourages advancement through research.”* Key informant 14, herbalist

## Discussion

The aim of the study was to explore collaboration between biomedical and traditional providers in mental health service delivery in Wakiso district, Uganda. The findings from this study highlight the importance of traditional healers in mental health service provision related to being perceived to be cheaper, more accessible, and an alternative to the formal mental health system. The study also established that barriers to the collaboration between traditional healers and biomedical practitioners such as non-inclusive mental health policies, perception that services by traditional healers are inferior to those by biomedical providers, and limited funding for mental health research. Although previous research has investigated the role of traditional healers in provision of mental health services in Uganda [34, 35], only limited studies [4, 18] have explored the views of traditional healers on their potential collaboration with biomedical healers to provide mental health services. This study therefore adds to the existing literature on provision of mental health services by traditional healers and their collaboration with biomedical professionals in Uganda. In addition, the findings reinforce evidence on the gaps and opportunities of collaboration between traditional healers and biomedical providers in mental health service provision globally.

Our study found that traditional healers were generally more accessible to the population due to several factors such as close proximity to the community, perceived more numbers compared to biomedical providers, and less bureaucracies involved with their services. Similar findings were reported in studies conducted in other African countries such as Kenya [36], Rwanda and South Africa [37] that found that communities widely consulted and sought treatment from traditional healers mainly due to their accessibility compared to biomedical providers. It is important to note that traditional healers may be less bureaucratic because they are currently largely unregulated in Uganda [5, 17] and other LMICs [37, 38].

Accessibility of providers of mental health services by the community is a key contributor to utilisation of health services. Green and Collucci suggest that one of the most effective ways of increasing accessibility for mental health services is through collaboration between traditional healers and biomedical providers. For example, collaboration would foster good working relationships where the more accessible traditional healers easily refer patients to biomedical practitioners hence increasing accessibility for biomedical health services [39]. However, power dynamics between the two categories of providers could hinder such collaboration as traditional medicine is usually considered inferior by various stakeholders including biomedical professionals [31]. Affordability also affects accessibility hence utilization of mental health services. Similarly in our study, traditional healers were perceived to be more affordable than biomedical providers hence their preference in the community. However, it should be noted that seeking services from biomedical professionals at public health facilities in Uganda is generally for free which is not the case in the private sector where user fees are paid. Other studies also found that the fact that traditional healers accepted both cash and non-monetary payments, as well as the opportunity to be paid in instalments increased their appeal to service users [36]. These reasons for community preference to traditional healers including accessibility and affordability could be used to improve mental health service provision within the health system.

From the study, mental health users had trust in the services provided by traditional healers. As a result, traditional healers were often the first point of mental health care as highlighted in a recent system review that showed that over 70% of individuals rely on traditional and faith based healers [40]. This was contrary to the views held by biomedical providers who participated in the study, as they believed that traditional healers were only a last option used when biomedical healers were not available. Evidence has shown that even in the presence of formal mental health care, community members may prefer traditional healers due to their ability to understand and interpret mental health in ways that are in alignment with community values and belief systems [41–43]. This is specifically important in mental health where social and cultural determinants play a major role. As traditional healers are the first point of care for many mental health patients, they should be supported to be more formally engaged in diagnosing mental health conditions including recognising signs and symptoms to ensure early and proper health seeking practices. Stakeholder engagement involving both traditional healers and biomedical providers could be employed to enhance the knowledge and capacity of traditional practitioners in mental health service delivery. This has been done for other diseases of public health concern, such as Ebola, which showed that traditional healers were key allies to curbing the disease in Ugandan communities [44]. In Nigeria and Ghana, a cluster randomised trial for a collaborative intervention between traditional and faith healers and primary health care workers was both effective and cost effective [45].

Participants in our study highlighted that the current mental health framework in Uganda hardly recognised traditional healers as mental health care providers. This implies that collaboration between the two providers of mental health services takes on an ad hoc and informal engagement [4]. WHO classifies collaboration between national mental health care systems and traditional healers as either integrative, inclusive or tolerant [28, 46]. Despite Uganda having made efforts to integrate traditional medicine, a unified working plan for mental health is still lacking. Based on Uganda’s policy, this collaboration is currently tolerant, implying that mental health care is entirely based on biomedical practice, and the laws and policies only tolerate a few traditional medicine practices. In this system, the laws are often poorly implemented or ignored. This is similar to most African countries which have tolerant mental health policies, and only a few countries such as South Africa, Rwanda [37], and Ghana [47] which have inclusive mental health policies. Unfortunately, no African country was found to have an integrative mental health policy which could be addressed in the future [48]. It is important to note that an inclusive mental health policy would be a foundation for defining the role of traditional healers in the mental health service structure. Although the mental health policy formulation and revision process in Uganda is generally slow, it is important to make progress towards inclusive policies which safeguard and provide benchmarks for good practices for all stakeholders in mental health. Fast-tracking the development and revision of mental health guidelines in Uganda, with clear avenues of collaboration between traditional healers and biomedical providers is needed.

From our study, limited funding allocated to mental health research was partly responsible for the hardly existent collaboration between biomedical practitioners and traditional healers. Recent studies have reported very little or no funding for mental health services and research in most African, Middle and Central Asian countries [49, 50]. Indeed, national mental health financing in Uganda currently covers a disproportionately small proportion of the necessary resources [17, 50]. In recent years, funding for mental health has increased, mainly from international funders and development partners due to increased importance of mental health on the global agenda. However, many international funders are more interested in funding biomedical research, hence limited funds are available for traditional medicine [51]. This therefore makes it difficult for traditional practitioners to test and standardize their practices and medicines. The lack of funding for traditional medicine can also be traced back to the colonial times were indigenous practices and customs were disbarred in favouritism for biomedical practices [4]. Therefore, there is need for countries, including Uganda, to increase priority for indigenous and local mental health research through allocating more funds and other non-monetary resources to traditional healers. In addition, governing bodies for those involved in traditional medicine, such as the National Drug Authority in Uganda, need to be supported to conduct and document their research.

A key strength of the study is that it considered the perspectives of traditional healers, biomedical practitioners and other stakeholders including policy makers. Contrary to previous studies [52, 53], this study did not seek to compare traditional healers to biomedical practitioner’s standards but rather to explore the perspectives of the two types of mental health providers which is also a study strength. One limitation of the study is that it only involved KIIs hence missed out the opportunity of getting perspectives from community members for example through group discussions. In addition, some of the KIIs were conducted virtually via Zoom due to the COVID-19 lockdown in Uganda at the time which could have affected the quality of the engagement with such participants. Indeed, interviews conducted virtually may not permit observation of non-verbal cues which are important in qualitative research. In addition, the use of snowball sampling could have introduced bias by participants recommending others from within their networks. Social desirability bias may have also been a concern especially among traditional healers, many of whom could have been aware that their practice was not fully regulated. The study was conducted in one district (Wakiso) in Central Uganda hence generalisation of the findings should be done with caution.

## Conclusion

Traditional healers were involved in the provision of mental health services in the community, many times as the first point of contact for patients seeking care. However, there was limited collaboration between traditional healers and biomedical practitioners. To increase collaboration between traditional healers and biomedical providers, there is need to recognise the contribution of traditional medicine, ensure mental health policies are more inclusive, and resources for mental health research are increased. Future research may pilot models for collaboration between the two categories of service providers.

## Supplementary Information

Below is the link to the electronic supplementary material.

Supplementary Material 1

Supplementary Material 2

## Data Availability

Data is provided within the manuscript or supplementary information files.

## Abbreviations

CHWs: Community health workers
KIIs: Key informant interview
LMICs: Low–and middle–income–countries
MOH: Ministry of Health
NGO: Non–governmental organisation
WHO: World Health Organization
