# Supplementary material for: The role of traditional healers and barriers to collaboration with biomedical providers in mental health service delivery in Wakiso district, Uganda: a qualitative study
Source: BMC Health Serv Res. 2026 Feb 19;26:403. doi: 10.1186/s12913-026-14170-5 (PMC13020018; doi:10.1186/s12913-026-14170-5)
Supplement: Supplementary file 1 — Supplementary Material 1 [file 12913_2026_14170_MOESM1_ESM.docx]

COLLABORATION BETWEEN BIOMEDICAL AND TRADITIONAL MENTAL HEALTH SERVICE PROVIDERS IN WAKISO DISTRICT, UGANDA

Codes

|  | Themes | Sub theme | Description | Files | References |
| --- | --- | --- | --- | --- | --- |
| 1 | Accessibility challenges |  | Delay in accessing MH services from BH | 0 | 0 |
|  |  | 1. Delay | Patients delay seeking assistance | 6 | 6 |
|  |  | 1. No hospital and MH services | No hospital and MH services in areas | 7 | 8 |
|  |  | 1. Parallel treatment | BH treatment is a standalone and not complemented with TM; Refugee communities have better services | 6 | 6 |
| 2 | Causes of Mental Health |  | Causes of Mental Health | 0 | 0 |
|  |  | 1. Alcohol and drug abuse | Alcohol and drug abuse and use of substances | 11 | 14 |
|  |  | 1. Stress-depression | MH diseases due to stress, anxiety, depression and hopelessness | 12 | 18 |
|  |  | 1. Wars-refugees | Impact of wars | 1 | 1 |
|  |  | 1. Witchcraft and ancestral spirits | MH diseases because of ancestral spirts and witchcraft | 9 | 16 |
| 2 | Challenges of the community |  |  | 0 | 0 |
|  |  | 1. Financial constraints | Financial constraints limiting access to drugs, transport and hospital care | 6 | 6 |
|  |  | - 1. No transport | Limited access to transportation means | 3 | 3 |
|  |  | 1. Lack of knowledge and awareness of MH services | Lack of knowledge and awareness of MH services | 8 | 11 |
|  |  | - 1. Misconception and beliefs | Misconception and beliefs on the origin and causes of MH | 16 | 24 |
|  |  | 1. Low adherence-patient expectations | Low adherence to medication and high patient expectation - immediate cures | 8 | 10 |
|  |  | 1. Stigma and family support | Stigma and family support | 4 | 5 |
| 3 | Challenges of health workers |  |  | 0 | 0 |
|  |  | 1. Drug stockouts | Drug stockouts | 12 | 13 |
|  |  | 1. Lack of collaboration TH and BMH | Lack of collaboration TH and BH | 18 | 31 |
|  |  | - 1. No collaboration | No collaboration between TH and BH | 10 | 10 |
|  |  | - - 1. Spite for TH | Spite for TH, not believing in their treatment method | 16 | 26 |
|  |  | 1. Lack of funding and research | Lack of funding and research; lack of facilitation | 9 | 15 |
|  |  | 1. Limited human resource | No MH specialists | 14 | 20 |
|  |  | 1. Not trained and inadequate training | BH are not trained | 6 | 7 |
| 4 | Common MH illness |  | Common MH diseases and illnesses in communities | 23 | 36 |
| 5 | MH providers |  | MH health providers; government and private, spiritualists, biomedical, families, religious leaders | 26 | 46 |
| 6 | Currently MH services offered |  | what is done; and services offered | 0 | 0 |
|  |  | 1. Collaboration and integration | collaboration and integration of BH and TH | 18 | 30 |
|  |  | - - 1. Reasons for collaboration | Same beliefs, prayer, saving of lifes | 5 | 6 |
|  |  | 1. Counselling and support services | Counselling and support services, giving hope and listening to patients; pre and post recovery services; rehabilitation | 17 | 35 |
|  |  | 1. Outreaches | Outreaches on MH | 6 | 10 |
|  |  | 1. Prayer | Use of prayer | 11 | 24 |
|  |  | 1. Referrals | Referrals | 29 | 113 |
|  |  | - - 1. BH to BH -VHT to HC | Biomedical healer to Biomedical healer and VHT to Biomedical Referrals | 17 | 30 |
|  |  | - - 1. BH to TH | Biomedical healer to Traditional healer Referrals | 3 | 4 |
|  |  | - - 1. TH to BH | Traditional healer to Biomedical healer Referrals | 15 | 40 |
|  |  | - - 1. TH to TH | Referral from Traditional healer to Traditional healer Referrals | 4 | 7 |
|  |  | 1. Specific days | Treatment on specific days/ MH clinic days | 2 | 2 |
|  |  | 1. TM as first treatment | Use of TM before any other form of treatment | 10 | 12 |
|  |  | 1. Treatment and medication |  | 18 | 42 |
|  |  | - - 1. Follow up and adherence to treatment | Provide follow up and service to enable adherence to medication | 4 | 6 |
|  |  | - - 1. Treatment | Treatment procedures in BM, assessing, medication | 12 | 14 |
|  |  | - - 1. Use of herbs | use of herbs; power or liquid extract medicine | 4 | 5 |
| 7 | Policy |  |  | 0 | 0 |
|  |  | 1. Importance of policies | importance of policies in addressing MH | 11 | 11 |
|  |  | 1. Knowledge on policies | Policies on MH | 19 | 41 |
|  |  | - 1. Knowledge on policies among BH | Policies and regulation for BM | 14 | 26 |
|  |  | - 1. Knowledge on policies among TH | MH regulations and guidelines among TH | 8 | 11 |
|  |  | 1. No policy and collaboration on MH | No policy on MH; Policies do not recognise TH or collaborations | 14 | 20 |
|  |  | 1. Not knowledgeable on policies | Participants who did not know MH policies | 7 | 7 |
| 8 | Solutions to good MH service provisions |  | Solutions to challenges of MH | 0 | 0 |
|  |  | 1. Collaboration |  | 28 | 83 |
|  |  | - 1. Bridge -Local leader | Use of local leaders and VHTs to bridge the gap between BH and TH | 4 | 5 |
|  |  | - 1. Collaboration and integration | Collaboration and integration of TH and BH | 17 | 23 |
|  |  | - 1. Will | Will of TB and BH to work together | 3 | 3 |
|  |  | - 1. Working relationship | working relationship | 17 | 29 |
|  |  | 1. Community sensitization and out reaches | community sensitization and out reaches to create awareness on MH and MH services; alignment of outreach services | 12 | 21 |
|  |  | - 1. Counselling services | counselling services; restoration of hope; overcome stigmatization | 4 | 5 |
|  |  | 1. Education and training | Education and training of both TH and BH; Combined training of TH and BH | 20 | 44 |
|  |  | 1. Funding and medical supplies | Funding and medical supplies; drug availability; transport; hospital infrastructure | 7 | 9 |
|  |  | 1. Increase of human resource | Increase of MH human resource | 8 | 9 |
|  |  | 1. Policy regulation | Policy regulation to protect MH providers and patients | 4 | 9 |
|  |  | 1. Recognition of TH | Recognition of TH | 11 | 19 |
|  |  | 1. Referral | Referral of patients between traditional healers and biomedical healers | 10 | 16 |
|  |  | - 1. VHT follow up | follow up of patients | 3 | 4 |
|  |  | 1. Research | Research on MH | 5 | 8 |
| 9 | Traditional medicine |  |  | 0 | 0 |
|  | 9a) Challenges of TH |  | Challenges of TH | 0 | 0 |
|  |  | 1. Depletion of raw materials | Depletion of raw materials like herbs | 2 | 2 |
|  |  | 1. Inadequate funds | challenges due to inadequate funds | 7 | 8 |
|  |  | 1. Inferiority complex |  | 1 | 1 |
|  |  | 1. Lack of recognition | TH are not recognised by government, and BH | 11 | 17 |
|  |  | 1. Safety at work | safety issues at work | 4 | 4 |
|  | 9b) Expertise and Calling |  | How TH joined the profession, powers used | 10 | 19 |
|  | 9c) Reasons and success TM |  | Reasons for TM | 0 | 0 |
|  |  | - 1. Accessibility | TH are more accessible | 6 | 8 |
|  |  | - - 1. Trust | Trust | 3 | 8 |
|  |  | - 1. Affordability | TH services are affordable | 3 | 3 |
|  |  | - 1. Effective treatment | Effective treatment | 7 | 9 |
|  |  | - - 1. Check up before diagnosis | Check up before diagnosis | 6 | 13 |
|  |  | - 1. Frustrations with modern medicine | Previous bad experience with modern medicine | 5 | 7 |
|  |  | - 1. Modern medicine can’t cure all diseases | Belief that modern medicine can’t cure all diseases | 5 | 9 |
|  |  | - 1. Word of mouth | TH get clients through word of mouth | 8 | 8 |
