# Supplementary material for: The role of traditional healers and barriers to collaboration with biomedical providers in mental health service delivery in Wakiso district, Uganda: a qualitative study
Source: BMC Health Serv Res. 2026 Feb 19;26:403. doi: 10.1186/s12913-026-14170-5 (PMC13020018; doi:10.1186/s12913-026-14170-5)
Supplement: Supplementary file 2 — Supplementary Material 2 [file 12913_2026_14170_MOESM2_ESM.pdf]

Appendix 1: Key informant interview guide for biomedical service providers

1. How would you describe your role in mental health care service delivery in your setting? *Probe: nature of services offered, types of mental health conditions seen / supported, number of years in service, challenges encountered, etc.*
2. Who are the various mental health care service providers in your setting? *Probe: different types of mental health providers at various health facility levels – both biomedical and traditional healers, etc.*
3. Is there any working relationship between you as a biomedical service provider and traditional healers regarding mental health service provision in your setting / community?
    a. If yes - please explain the nature of the existing relationship.
        i. What facilitates this relationship? e.g. policies, community leadership, etc.
        ii. What can be done to enhance / strengthen this relationship?
    b. If no - why? What are the barriers to a good working relationship between you and non-biomedical / traditional mental health care providers?
        i. What can be done to facilitate such a collaboration?
        ii. Under what circumstances (if at all) would you be willing to collaborate with non-biomedical / traditional / other mental health care providers?
4. Are you aware of any existing policies and guidelines governing the provision of mental health care services among biomedical service providers? If so, provide more details.
5. What are your perceptions regarding existing policies and legislation in relation to collaboration between biomedical service providers and non-biomedical / traditional mental health care providers?
6. What can be done to promote your visibility and contribution to mental health service delivery in your setting?
7. Do you have any final insights on mental health service delivery particularly regarding biomedical service providers and traditional healers?

Thank you for your time.

Appendix 2: Key informant interview guide for policy makers / other national level stakeholders

1. Who are the providers of mental health care services across the country? *Probe: biomedical service providers, traditional healers, others such as religious leaders, etc; describe their roles in mental health service provision; types of mental health conditions seen / supported by the various providers; etc.*
2. Do you recognise traditional healers as mental health care providers?
    i. If yes - how do the existing policies / guidelines define traditional healers? Briefly describe their roles
    ii. If no - please explain why. What can be done to promote their recognition and contribution to mental health service provision in the country?
3. In your opinion, is collaboration between biomedical and traditional / other non-biomedical mental health care providers feasible? Please explain.
4. Are you aware of any existing collaboration between biomedical service providers and non-biomedical mental health care providers / traditional healers concerning mental health service provision across the country? If yes, please cite examples and describe the nature of such collaboration.
5. Are there existing policies / guidelines that advocate for collaboration between biomedical and traditional mental health care providers?
    a. If yes, please provide more details.
    b. If no, can formulation of policies/guidelines better facilitate collaboration between biomedical and non-biomedical / traditional mental health care providers? Kindly provide more details.
6. Do you have any final insights on mental health service delivery particularly regarding biomedical service providers and traditional healers?

Thank you for your time.

Appendix 3: Key informant interview guide for traditional healers (English)

1. How would you describe your role in mental health care delivery in your community? *Probe: nature of services offered, types of mental health conditions seen / supported, number of years in service, challenges encountered, etc.*
2. Who are the mental health care providers in your setting? *Probe: different types of mental health providers – both biomedical and traditional, religious leaders, any others, etc.*
3. Is there any working relationship between you and biomedical or other mental health care providers in your community?
    a. If yes - please explain the nature of the existing relationship.
        i. What facilitates this relationship? e.g. policies, community leadership, etc.
        ii. What can be done to enhance / strengthen this relationship?
    b. If no - why? What are the barriers / gaps to a good working relationship between you and biomedical or other mental health care providers?
        i. What can be done to facilitate a better collaboration?
        ii. Under what circumstances (if at all) would you be willing to collaborate with biomedical or other mental health care providers?
4. Are you aware of any existing policies and guidelines governing the provision of mental health care services among traditional healers? If so, provide more details.
5. What are your perceptions regarding existing policies and legislation in relation to collaboration between biomedical service providers and non-biomedical / traditional mental health care providers?
6. What can be done to promote your visibility and contribution to mental health service delivery in the community?
7. Do you have any final insights on mental health service delivery particularly regarding biomedical service providers and traditional healers?

Thank you for your time.

Appendix 4: Key informant interview guide for Community Health Workers (English)

1. Do you play any role concerning mental health care service delivery in your setting? *Probe: nature of services offered, types of mental health conditions seen / supported, number of years in service, challenges encountered, etc.*
2. Who are the various mental health care service providers in your community? *Probe: different types of mental health providers at various health facility levels – both biomedical and traditional healers, etc.*
3. Is there any working relationship between biomedical service providers and traditional healers regarding mental health service provision in your setting / community?
    a. If yes - please explain the nature of the existing relationship.
        i. What facilitates this relationship? e.g. policies, community leadership, etc.
        ii. What can be done to enhance / strengthen this relationship?
    b. If no - why? What are the barriers to a good working relationship between you and non-biomedical / traditional mental health care providers?
        i. What can be done to facilitate such a collaboration?
        ii. Under what circumstances (if at all) would you be willing to collaborate with non-biomedical / traditional / other mental health care providers?
4. What challenges do community members face regarding seeking services for mental health conditions in your area?
5. What can be done to promote your visibility and contribution to mental health service delivery in your setting?
6. Do you have any final insights on mental health service delivery particularly regarding biomedical service providers and traditional healers?

Thank you for your time.
